# Paradoxical impairment of angiogenesis, endothelial function and circulating number of endothelial progenitor cells in DPP4-deficient rat after critical limb ischemia

**DOI:** 10.1186/scrt181

**Published:** 2013-03-21

**Authors:** Cheuk-Kwan Sun, Steve Leu, Jiunn-Jye Sheu, Tzu-Hsien Tsai, Hsin-Chin Sung, Yung-Lung Chen, Sheng-Ying Chung, Sheung-Fat Ko, Hsueh-Wen Chang, Hon-Kan Yip

**Affiliations:** 1 Department of Emergency Medicine, E-Da Hospital, I-Shou University, No.1, Yida Road, Jiaosu Village, Yanchao District, Kaohsiung 82445, Taiwan; 2 Center for Translational Research in Biomedical Sciences, Kaohsiung Chang Gung Memorial Hospital and Chang Gung University College of Medicine, 123 Dapi Road, Niaosung District, Kaohsiung 83301, Taiwan; 3 Division of Cardiovascular Surgery, Department of Surgery, Kaohsiung Chang Gung Memorial Hospital and Chang Gung University College of Medicine, 123 Dapi Road, Niaosung District, Kaohsiung 83301, Taiwan; 4 Division of Cardiology, Department of Internal Medicine, Kaohsiung Chang Gung Memorial Hospital and Chang Gung University College of Medicine, 123 Dapi Road, Niaosung District, Kaohsiung 83301, Taiwan; 5 Department of Anatomy, Chang Gung University, 259 Wen-Hwa 1st Road, Kwei-Shan, Tao-Yuan 333, Taiwan; 6 Department of Radiology, Kaohsiung Chang Gung Memorial Hospital and Chang Gung University College of Medicine, 123 Dapi Road, Niaosung District, Kaohsiung 83301, Taiwan; 7 Department of Biological Sciences, National Sun Yat-Sen University, 70 Lienhai Road, Kaohsiung 80424, Taiwan

## Abstract

**Introduction:**

We hypothesized that dipeptidyl peptidase-IV (DPP4) may impair angiogenesis, endothelial function, and the circulating number of endothelial progenitor cells (EPC) in a model of critical limb ischemia (CLI) through ligating the left femoral artery using DPP4-deficient rats.

**Methods:**

Adult male DPP4-deficient (DPP4^D^) rats (*n *= 18) were equally divided into CLI only (DPP4^D^-CLI) and CLI treated by granulocyte colony-stimulating factor (GCSF) (DPP4^D^-CLI-GCSF). For comparison, age-matched wild-type (WT) Fischer 344 rats (*n *= 18) were randomized into two groups receiving identical treatment compared to their DPP4-deficient counterparts and labeled as WT-CLI (*n *= 9) and WT-CLI-GCSF (*n *= 9), respectively.

**Results:**

The circulating number of EPCs (CD31+, CD34+, CD133, C-kit+) was significantly lower in DPP4-deficient than in WT rats on post-CLI days 1 and 4 (all *P *< 0.01). The ratio of ischemia/normal blood flow was remarkably lower in DPP4^D^-CLI-GCSF rats than in WT-CLI-GCSF animals on post-CLI Day 14 (all *P *< 0.01). Protein expressions of pro-angiogenic factors (endothelial nitric oxide synthase (eNOS), CXCR4, SDF-1α, vascular endothelial growth factor (VEGF)) were remarkably higher in WT-CLI than in DPP4^D^-CLI rats, and higher in WT-CLI-GCSF than in DPP4^D^-CLI-GCSF animals (all *P *< 0.01). Moreover, the numbers of small vessel in the ischemic area were substantially higher in WT-CLI-GCSF than in DPP4^D^-CLI-GCSF rats (*P *< 0.001). Furthermore, vasorelaxation and nitric oxide production of the normal femoral artery were significantly reduced in DPP4-deficient than in WT Fischer rats (all *P *< 0.01).

**Conclusions:**

Contrary to our hypothesis, DPP4-deficient rats were inferior to age-matched WT Fischer rats in terms of angiogenesis, endothelial function, circulating EPC number and response to GCSF, suggesting a positive role of DPP4 in maintaining vascular function and tissue perfusion in this experimental setting.

## Introduction

Abundant data have demonstrated that endothelial dysfunction (ED) is a systemic process that is the first step in the pathogenesis of atherosclerosis and atherosclerotic plaque progression [1-4]. A strong association between ED and risk factors of coronary artery disease (CAD) has been well documented [3,5-8]. Additionally, clinical observational studies have revealed that accumulative CAD risk factors are predictive of a decreased circulating number of endothelial progenitor cells (EPCs) [9-12]. Besides, not only has a reduction in circulating levels of EPCs been previously shown to be strongly correlated to future cardiovascular events and the progression of atherosclerosis in patients with CAD [11-13], but it has also been found to be predictive of future recurrent ischemic stroke [14]. On the other hand, an increase in circulating levels of EPCs [10,14-17] or therapy using vascular stem/progenitor cells [18,19] is believed to play a crucial role in vascular endothelial repair, angiogenesis and reduction of the sequelae of ischemic syndrome.

Stromal cell-derived factor (SDF)-1α, a chemokine, plays a key role in the mobilization of EPCs from bone marrow to circulation and ischemic area for angiogenesis [20,21]. In addition, SDF-1α, a natural substrate of dipeptidyl peptidase IV (DPP4) enzyme (CD26/DPP4 is a membrane-bound extracellular peptidase), is degraded by this enzyme in circulation [20,22]. Moreover, experimental study [20] has previously demonstrated that inhibition of DPP4 activity by angiotensin converting enzyme inhibitor (ACEI) increased circulating concentration and prolonged the biological half-life of SDF-1α which, in turn, enhanced circulating number of EPC in ischemic condition. Consistently, clinical observational studies have shown that inhibition of DPP4 activity by sitagliptin, an oral hypoglycemic agent, can increase circulating EPC levels in patients with type II diabetes mellitus [23]. Furthermore, previous studies have demonstrated that granulocyte colony-stimulating factor (GCSF) enhances the mobilization of stem cells and EPCs from bone marrow into circulation [24,25]. Therefore, through induction of critical limb ischemia (CLI), this study tested the hypothesis that male DPP4-deficient rats (DPP4 mutant of Fischer 344, that is, deficiency of DPP4 enzyme activity) may have a higher circulating number of EPCs and better preserved endothelial function, angiogenesis capacity and perfusion in the ischemic area compared with age-matched wild-type male Fischer 344 rats. This study further investigated whether GCSF treatment contributes to an enhancement of these biomarkers, thereby increasing blood flow to the ischemic area.

## Methods

### Ethics

All animal experimental procedures were approved by the Institute of Animal Care and Use Committee of Kaohsiung Chang Gung Memorial Hospital (No. 2009091501) and performed in accordance with the Guide for the Care and Use of Laboratory Animals (NIH publication No. 85-23, National Academy Press, Washington, DC, USA, revised 1996).

### Animal model of critical limb ischemia

Seven-month-old male DPP4-deficiency (DPP4^D^) rats (420 to 450 gm) (*n *= 18) (BioLASCO Taiwan Co., Ltd., Yilan, Taiwan) were divided into CLI without treatment (DPP4^D^-CLI, *n *= 9) and CLI treated with GCSF (100.0 μg/kg/day for five consecutive days after CLI procedure), (DPP4^D^-CLI-GCSF, *n *= 9). The GCSF dosage utilized in this study was based on previous reports [26,27]. For comparison, age-matched wild-type (WT) adult male Fischer 344 rats (Charles River Technology, BioLASCO Taiwan Co., Ltd., Yilan, Taiwan) were similarly divided into CLI without treatment (WT-CLI, *n *= 9) and CLI treated with GCSF (100.0 μg/kg/day) for five consecutive days after the CLI procedure (WT-CLI-GCSF, *n *= 9).

Another two groups of animals, including age-matched adult male DPP4-deficient rats (*n *= 6) and WT Fischer 344 rats (*n *= 6) without receiving CLI procedure or any treatment, were used as normal controls, and labeled as DPP4^D^-NC and WT-NC in the current study, respectively.

The procedure of CLI was as previously described [28]. Under sterile conditions, the left femoral artery, small arterioles and circumferential femoral artery were exposed and ligated over their proximal and distal portions before removal. The rats were sacrificed on Day 14 after CLI induction and the left quadriceps muscle were collected for individual study.

### Flow cytometric quantification of endothelial progenitor cells based on surface markers

To identify serial changes in the circulating number of EPCs, peripheral blood (1.0 mL each time) was drawn from the tail vein into a vacutainer containing 3.8% buffered sodium heparin in animals undergoing CLI prior to the procedure and at one hour and on days 1, 4, and 14 after the CLI procedure. Mononuclear cells (MNCs) were then isolated by density-gradient centrifugation of Ficoll 400 (Ficoll-Plaque™ plus, Amersham Biosciences, Piscataway, NJ, USA) as previously described [12,14,17].

To identify the population of EPCs prior to and following the CLI procedure, MNCs were immunostained for 30 minutes on ice with the following antibodies: PE-conjugated antibodies against CD133 (BD Pharmingen, Franklin Lakes, NJ, USA) and CD34 (BD Pharmingen); Fluorescein isothiocyanate (FITC)- against c-kit (BD Pharmingen); Monoclonal antibodies against CD31 (Abcam, Cambridge, MA, USA). Cells labeled with non-fluorescence-conjugated antibodies were further incubated with Alexa Fluor 488-conjugated antibodies specifically against mouse or rabbit IgG (Invitrogen Co., Ltd., Carlsbad, CA, USA). Isotype-identical antibodies (IgG) served as controls. Flow cytometric analyses were performed by utilizing a fluorescence-activated cell sorter (Beckman Coulter FC500 flow cytometer, Beckman Coulter Inc., Brea, CA, USA). The detailed procedure of flow cytometric analysis has been depicted in our recent report [12,14,17].

### Measurement of femoral arterial contractility and nitric oxide release on D-galactose challenge

To elucidate the effect of D-galactose challenge on vascular function (that is, vasoconstriction (phenylephrine-stimulating response), vasorelaxation (acetylcholine-stimulating response) and basal nitric oxide (NO) release (L-NAME-mediated blockade)) [29], both DPP4-deficient rats (*n *= 12) and WT Fischer rats (*n *= 12) without receiving any other treatment were divided into physiological saline-treated groups (2.0 mL/day intravenous injection, *n *= 6 from each group) and D-galactose-loading groups (500 mg/kg/day intravenous injection, *n *= 6 from each group) for 10 weeks.

At the end of the study, the right femoral artery was isolated from each of the rats, cleaned, and cut into slices of 2 mm in length for evaluating the contractile and relaxant response as previously reported [30] with some modifications. Briefly, femoral arterial rings were carefully mounted on an isometric force transducer (XDFT05, Singa Biotechnology Ltd., Kaohsiung, Taiwan) with a tension of 1.8 g, and placed in an organ chamber filled with Krebs solution (NaCl, 99.01 mM; KCl, 4.69 mM; CaCl_2_, 1.87 mM; MgSO_4_, 1.20 mM; K_2_HPO_4_, 1.03 mM; glucose, 11.1 mM) maintained at pH 7.4 and bubbled with 95%O_2_-5%CO_2_. After an equilibration of 40 minutes, 1 μM of phenylephrine (PE) was added to the organ chamber for the assessment of contractile activity, and then 30 μM of acetylcholine (ACh) was added to assess the endothelial integrity. After washing and a re-equilibration for 30 minutes, a cumulative PE dose (from 1 nM to 1 μM) was added to the organ chamber to obtain a concentration-dependent contractile curve, and then sodium nitroprusside (30 μM) was added to the organ chamber to obtain a relaxant response. After washing and a re-equilibration for 20 minutes, 30 μM of ACh was added into the organ chamber followed by 1 μM of PE to evaluate the endothelium-dependent vasorelaxant response. Then PE (1 μM)-induced vasocontractile response was assessed again in the presence of L-NAME (100 μM) pre-treatment for 30 minutes. All data were acquired and analyzed using the XctionView system (XctionView, Singa, Taiwan).

### Protocol for assessment of arterial basal NO release on D-galactose overload

Vascular basal nitric oxide release was calculated as the percentage of difference between PE-induced vasocontractile response in the absence and presence of L-NAME according to our previous study [31].

### Measurement of blood flow with Laser Doppler

Rats were anesthetized by inhalation of 2.0% isoflurane prior to CLI induction and on days 2 and 14 after CLI induction prior to be sacrificed (*n *= 9 for each group). The rats were placed in a supine position on a warming pad at 37°C. After being shaved over bilateral hind limbs and inguinal areas, blood flow was surveyed by a Laser Doppler scanner (moorLDLS, Moor instruments Ltd., Devon, UK). The ratio of blood flow of left hind limb (ischemic) to that of the right side (normal) was computed.

### Western blot analysis

Equal amounts (10 to 30 mg) of protein extracts from ischemic quadriceps of the animals (*n *= 6 for each group) were loaded and separated by SDS-PAGE using 12% acrylamide gradients. The membranes were incubated with monoclonal antibodies against vascular cell adhesion molecule CXCR4 (1:1,000, Abcam), vascular endothelial growth factor (VEGF) (1:1,000, Abcam), stromal cell-derived growth factor (SDF)-1α (1:1,000, Cell Signaling Technology, Inc., Danvers, MA, USA), and endothelial nitric oxide synthase (endothelial nitric oxide synthase (eNOS)) (1:1,000, Abcam). Signals were detected with horseradish peroxidase (HRP)-conjugated goat anti- mouse or goat anti-rabbit IgG. Proteins were transferred to nitrocellulose membranes and followed by incubation with secondary antibody solution (1:300) for one hour at room temperature. The washing procedure was repeated eight times within 40 minutes. Immunoreactive bands were visualized by enhanced chemiluminescence (ECL; Amersham Biosciences) which was then exposed to Biomax L film (Kodak Ltd., Rochester, NY, USA). For quantification, ECL signals were digitized using Labwork software (UVP Inc., Waltham, MA, USA).

### Immunofluorescent (IF) staining

IF staining was performed for the examinations of CD31+, von Willebrand factor (vWF)+, and CXCR4+ cells (*n *= 6 for each group) using respective primary antibodies based on our recent study [28]. Irrelevant antibodies were used as controls in the current study.

### Vessel density in limb ischemic area

Immunohistochemical (IHC) staining of blood vessels was performed (*n *= 6 for each group) with α-SMA (1:400) as primary antibody at room temperature for one hour, followed by washing with PBS thrice according to our recent study [28]. Three sections of quadriceps were analyzed in each rat. For quantification, three randomly selected HPFs (x100) were analyzed in each section. The mean number per HPF for each animal was then determined by summation of all numbers divided by nine.

### Determination of SDF-1α level in bone marrow (BM) and circulation

To determine SDF-1α levels in BM and circulation at 18 h after the CLI procedure, another 12 DPP4-deficient rats and 12 WT Fischer rats (that is, 6 in each group) were utilized for this study. These rats were sacrificed at 18 h after the procedure and the serum from both BM and circulation was collected for determining SDF-1α level using ELISA analysis. Serum SDF-1α concentration was assessed by duplicated determination with a commercially available ELISA kit (B & D Systems, Inc., Minneapolis, MN, USA). The lower detection limit was 0.156 ng/mL.

### Statistical analysis

Quantitative data are expressed as means ± SD. Statistical analysis was adequately performed by ANOVA followed by Bonferroni multiple-comparison *post hoc *test. SAS statistical software for Windows version 8.2 (SAS Institute, Cary, NC, USA) was used in this study. A probability value < 0.05 was considered statistically significant.

## Results

### Flow cytometric quantification of serial changes in circulating endothelial progenitor cell surface markers

**Figure 1:**
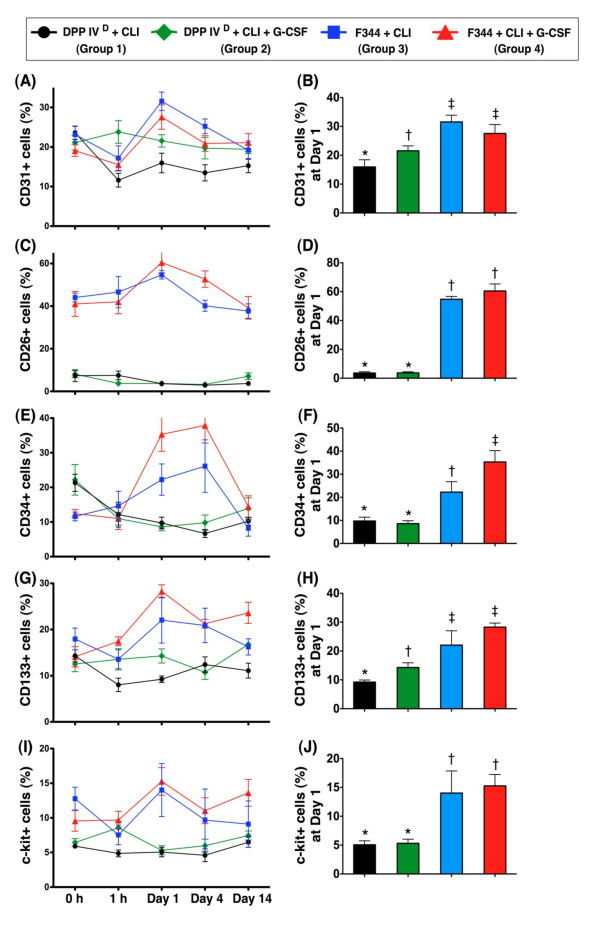
**Changes in number of endothelial progenitor cells (EPCs) (CD31+, CD34+, CD133+, C-kit+) and CD26+ cells at different time points**. **A, E, G, I) **Numbers of EPC at 0 and 1 h and days 1, 4 and 14 after induction of critical limb ischemia (CLI). Except for CD34-EPCs, the peak levels of EPCs in Fischer 344 rats appeared on Day 1 **(B, F, H, J) **after CLI. Notably higher levels of EPCs in wild-type (WT) Fischer rats compared to DPP4-deficient rats at days 1 and 4 following CLI. By Day 14, CD31-EPCs and C-kit-EPCs remained higher in CLI WT rats treated with granulocyte-colony stimulating factor (GCSF) than in DPP4-deficient rats regardless of GCSF treatment. **C, D) **Persistently lower number of CD26+ cells in DPP4-deficient animals than in WT Fischer rats. * vs. other groups, *P *< 0.001. All statistical analyses using one-way ANOVA, followed by Tukey's multiple comparison procedure. Symbols (*, †, ‡) indicate significance (at 0.05 level). DPP4^D ^= DPP4-deficient (*n *= 9 per group).

Flow cytometric analyses showed no difference in circulating CD31+ cells between DPP4-deficient and WT Fischer rats prior to the CLI procedure (Figure 1A). Similarly, the number of CD31+ cells did not significantly differ between WT-CLI rats with and without GCSF treatment at time points of 1 h, days 1, 4 and 14 after CLI induction. However, the number of CD31+ cells was significantly higher in the DPP4^D^-CLI-GCSF group than in other groups at 1 h, and significantly higher than in the DPP4^D^-CLI group on days 1, 4 and 14 after CLI. On the other hand, CD31+ was notably increased in WT-CLI rats with or without GCSF treatment than in DPP4^D^-CLI animals with or without GCSF treatment on Day 1, and significantly increased in WT-CLI rats (that is, with or without GCSF treatment) than in the DPP4^D^-CLI group on Day 4. One important finding is that the peak level of this biomarker in circulation appeared on Day 1 after the CLI procedure in WT Fischer rats, but not in their DPP4-deficient counterparts (Figure 1A).

The circulating number of CD26+ cells (that is, an index of CD26/DPP4 activity) was substantially lower in DPP4-deficient rats with or without GCSF treatment than in WT rats with or without GCSF treatment prior to and at 1 h, days 1, 4 and 14 after the CLI procedure, but GCSF treatment made no significant difference in the DPP4-deficient and WT rats at these time points (Figure 1C). The peak level of this biomarker was on Day 1 in WT rats with or without GCSF treatment after the CLI procedure (Figure 1C).

Prior to CLI induction, the circulating number of CD34+ cells was significantly higher in DPP4-deficient rats than in their WT counterparts (Figure 1E). On the other hand, this biomarker was remarkably higher in the WT-CLI-GCSF rats than in other groups, notably higher in WT-CLI rats without GCSF treatment than in DPP4-deficient rats with or without GCSF administration that showed no difference among themselves on days 1 and 4 after CLI induction. However, this parameter was similar among the four groups on Day 14 after the procedure. Interestingly, the peak level of circulating CD34+ cells appeared at the time point of Day 4 in WT rats with or without GCSF treatment after the CLI procedure (Figure 1E).

The circulating number of CD133+ cells did not differ among the four groups prior to CLI induction (Figure 1G). This biomarker was lower in the DPP4^D^-CLI group than in other groups that showed no significant difference among themselves at 1 h after the procedure. Additionally, it was significantly higher in WT rats with or without GCSF treatment compared with their DPP4-deficient counterparts (that is, with or without GCSF treatment) at the time points of days 1 and 4 after CLI. Furthermore, this biomarker was notably higher in DPP4^D^-CLI rats with GCSF than in those without, and higher in WT-CLI rats with GCSF than in those without on Day 1 (Figure 1H), but it did not differ between DPP4^D^-CLI rats with and without GCSF treatment or between WT rats with and without GCSF treatment on Day 4 after the CLI procedure. Moreover, this biomarker was highest in the WT-CLI-GCSF group but lowest in the DPP4^D^-CLI animals on post-CLI Day 14. Consistent with the finding of circulating levels of CD31+ cells, the peak level of circulating CD133+ cells also appeared in WT rats with and without GCSF treatment on Day 1 after CLI induction (Figure 1G).

The circulating number of C-kit+ cells did not differ between DPP4-deficient rats with and without GCSF treatment as well as between WT rats with and without GCSF treatment, but it was significantly higher in WT than in DPP4-deficient animals prior to CLI induction (Figure 1I). Moreover, this biomarker was notably higher in DPP4-deficient and WT rats having received GCSF treatment, than in DPP4^D^-CLI rats, but there was no significant difference between DPP4^D^-CLI and WT animals without GCSF at 1 h after the procedure. Furthermore, this circulating biomarker was remarkably higher in WT than in DPP4-deficient rats, but it showed no difference between DPP4-deficient rats with and without GCSF treatment, as well as between WT rats with and without GCSF treatment on post-CLI Day 1 (Figure 1J). On the other hand, it remained significantly higher in the WT-CLI-GCSF group than in the DPP4-deficient rats with or without GCSF treatment on days 4 and 14 after the CLI procedure. Once again, we found that the peak level of circulating C-kit+ cells still appeared on Day 1 in WT rats with or without GCSF treatment after the CLI procedure (Figure 1I). Suppressed numbers of CD31+, CD34+, CD133+ and C-kit+ cells, therefore, implied that DPP4-deficient rats had notably reduced ability of mobilizing EPCs into the circulation in response to CLI regardless of the presence or absence of G-CSF.

### Laser Doppler analysis of blood flow

**Figure 2:**
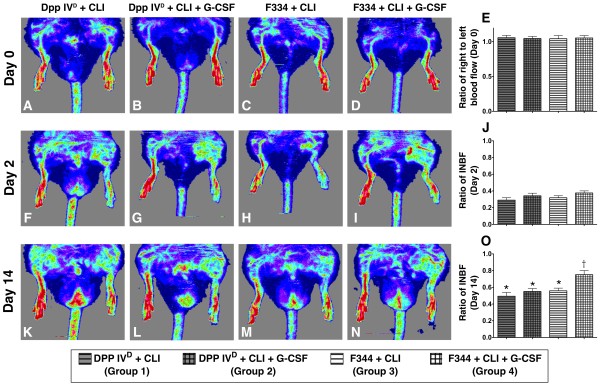
**Laser Doppler scanning of blood flow over hind limbs on Day 14 after critical limb ischemia (CLI)**. **A to D) **Normal hind limb blood flow prior to CLI procedure. **F to I) **Markedly reduced blood flow on left side on Day 2 after CLI, validating the CLI model. **K to N) **Notably increased blood flow in CLI wild-type (WT) Fischer rats treated with GCSF **(N) **compared to that in other groups by Day 14 after CLI. **E, J, O) **Ratio of ischemic/normal blood flow (INBF) prior to (that is, Day 0) and on days 2 and 14 after CLI. **J) **Notably lower INBF ratio in all groups by Day 2 after CLI compared with normal condition. **O) **Remarkably higher INBF ratio in the WT-CLI-GCSF group than in other groups by Day 14 after CLI procedure and without significant difference among groups 1, 2 and 3. Statistical analysis by one-way ANOVA. * vs. †, *P *< 0.01. Symbols (*, †) indicate significant difference (at 0.05 level) by Tukey's multiple comparison procedure (*n *= 9 per group).

Laser Doppler scanning demonstrated no difference in the ratio of ischemic/normal blood flow (INBF) among the four groups prior to (that is, Day 0) CLI induction (Figure 2A-E). Similarly, there was no significant difference among the four groups on post-CLI Day 2 (Figure 2F-J). Compared with their respective INBF on Day 0, marked reduction was noted in all four groups on post-CLI Day 2. The decrease in all four groups was still significant 14 days after CLI induction (Figure 2K-O) compared to that on Day 0, although the WT-CLI-GCSF group showed only a minor reduction compared to its level on Day 0. On post-CLI Day 14, substantially higher INBP was noted in the WT-CLI-GCSF group compared to the other three groups that showed no significant difference among themselves (Figure 2K-O).

### Protein expressions of pro-angiogenic markers (eNOS, VEGF, CXCR4 and SDF-1α) by post-CLI Day 14

**Figure 3:**
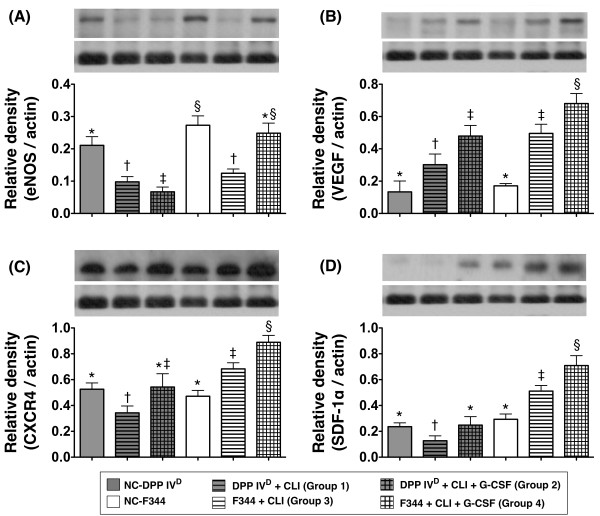
**Protein expressions of pro-angiogenic factors in ischemic skeletal muscle on Day 14 following CLI induction**. **A) **In DPP4-deficient animals, notably higher eNOS expression in normal control (DPP4^D^-NC) than in DPP4^D^-CLI and DPP4^D^-CLI-GCF. * vs. †, *P *< 0.05. In wild-type (WT) Fischer rats, notably higher in normal controls (WT-NC) and WT-CLI-GCSF rats than in WT-CLI animals without difference between WT-NC and the WT-CLI-GCSF group. † vs. ‡, *P *< 0.01. **B) **In DPP4-deficient animals, notably higher VEGF expression in those after CLI induction with GCSF treatment than in those without and the non-CLI group (DPP4^D^-NC), and higher in DPP4-deficient rats after CLI induction than in DPP4^D^-NC. * vs. † vs. ‡, *P *< 0.01. In WT Fischer rats, significantly higher in WT-CLI-GCSF group than in WT-CLI and WT-NC, higher in WT-CLI than in WT-NC. * vs. † vs. ‡, *P *< 0.005. **C) **In DPP4-deficient animals, significantly higher CXCR4 expression level in DPP4^D^-CLI-GCSF and DPP4^D^-NC than in DPP4^D^-CLI, without difference between the former two groups. * vs. †, *P *< 0.03. In WT Fischer rats, remarkably higher in WT-CLI-GCSF than in WT-CLI and WT-NC, and higher in WT-CLI than in WT-NC. * vs. † vs. ‡, *P *< 0.01. **D) **In DPP4-deficient animals, significantly lower stromal cell-derived factor (SDF)-1α protein expression in DPP4^D^-CLI than in DPP4^D^-NC and DPP4^D^-CLI-GCSF group, without difference between the latter two groups. * vs. †, *P *< 0.04. In WT Fischer rats, markedly increased in WT-CLI-GCSF than in WT-CLI and WT-NC, and higher in WT-CLI than in WT-NC. * vs. † vs. ‡, *P *< 0.001. A-D) Statistical analysis by one-way ANOVA. * vs. other groups, *P *< 0.001. Symbols (*, †, ‡, §) indicate significant difference (at 0.05 level) by Tukey's multiple comparison procedure (*n *= 6 per group).

In DPP4-deficient animals, Western blot analysis demonstrated notably lower eNOS protein expression in DPP4^D^-CLI rats with or without GCSF treatment than that in their counterparts without CLI (DPP4^D^-NC) (Figure 3A). In WT Fischer rats, eNOS protein expression was also more notably reduced in the WT-CLI group than in the non-CLI group (WT-NC) (Figure 3A). Moreover, it was remarkably higher in the WT-CLI-GCSF group than in other CLI groups, but there was no significant difference between the WT-CLI-GCSF and WT-NC animals.

The protein expression of VEGF was significantly higher in DPP4-deficient rats with GCSF treatment compared to those without and the DPP4^D^-NC group, and notably higher in the WT animals with GCSF than in those without and the WT-NC group (Figure 3B). In addition, it was also higher in the DPP4^D^-CLI group than in the DPP4^D^-NC group and significantly higher in the WT-CLI group than in the WT-NC group (Figure 3B). Interestingly, it was also significantly higher in WT-CLI than in DPP4^D^-CLI rats and notably higher in WT-CLI-GCSF than in WT-CLI animals (Figure 3B).

In DPP4-deficient animals, CXCR4 protein expression was similar between DPP4^D^-NC and the DPP4^D^-CLI-GCSF group (Figure 3C). However, it was notably reduced in DPP4^D^-CLI group than in DPP4^D^-CLI-GCSF and DPP4^D^-NC groups. On the other hand, in WT Fischer rats, it was notably higher in WT-CLI-GCSF than in WT-CLI and the WT-NC group, and significantly increased in the WT-CLI group compared with that in the WT-NC animals (Figure 3C).

In DPP4-deficient rats, the protein expression of SDF-1α was significantly lower in the DPP4^D^-CLI group than in DPP4^D^-NC and DPP4^D^-CLI-GCSF groups, but it showed no difference between the DPP4^D^-NC and DPP4^D^-CLI-GCSF group (Figure 3D). In WT Fischer rats, it was remarkably higher in WT-CLI-GCSF than in WT-CLI and WT-NC animals, and significantly higher in WT-CLI than in WT-NC group (Figure 3D). Of particular importance is that it was significantly higher in WT rats with or without GCSF treatment than in DPP4-deficient rats with or without GCSF treatment after CLI induction.

### IF and IHC staining of ischemic quadriceps on Day 14 after CLI procedure

**Figure 4:**
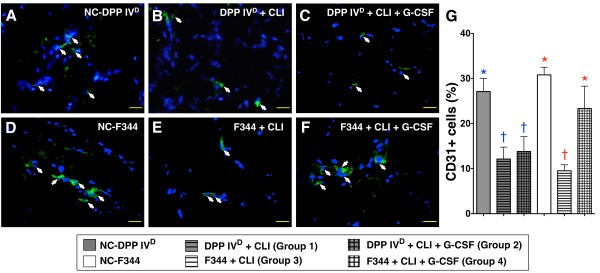
**Distribution of CD31+ cells in ischemic skeletal muscle on Day 14 following CLI induction**. **A to F) **Immunofluorescent staining of CD31+ cells (white arrows) in ischemic skeletal muscle on post-CLI Day 14 with nuclei being counter-stained with DAPI (blue) (400x, *n *= 6 per group). **G) **In DPP4-deficient animals, significantly lower number of CD31+ cells in those with or without GCSF treatment than in DPP4^D^-NC, without difference between the former two groups. * vs. †, *P *< 0.001. In WT Fischer animals, notably lower cell number in WT-CLI than in WT-NC and WT-CLI-GCSF, without difference between the latter two groups. * vs. †, *P *< 0.0001. For comparison among the six groups, statistical analysis with one-way ANOVA. * vs. other groups, *P *< 0.0001. Symbols (*, †) indicate significant difference (at 0.05 level) by Tukey's multiple comparison procedure.

**Figure 5:**
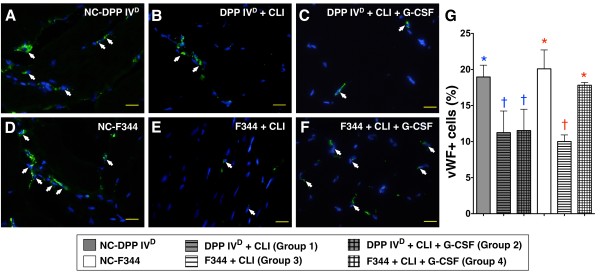
**Distribution of von Willebrand factor (vWF)+ cells in ischemic skeletal muscle on Day 14 following CLI induction**. **A to F) **Immunofluorescent staining of cells positive for von Willebrand factor (vWF) (white arrows), an indicator of endothelial cells, in ischemic area of each group (*n *= 6) on post-CLI with nuclei being counter-stained with DAPI (blue) (400x, *n *= 6 per group) (400x). **G) **In DPP4-deficient animals, significantly lower number of vWF+ cells in those with or without GCSF treatment than in DPP4^D^-NC, with no difference between the former two groups. * vs. †, *P *< 0.01. In WT Fischer rats, notably lower number of positively-stained cells in WT-CLI than in WT-NC and WT-CLI-GCSF, without difference between the latter two groups. * vs. †, *P *< 0.001. For comparison among the six groups, statistical analysis by one-way ANOVA. * vs. other groups, *P *< 0.001. Symbols (*, †) indicate significant difference (at 0.05 level) by Tukey's multiple comparison procedure.

In DPP4-deficient animals, IF staining of quadriceps demonstrated significantly higher numbers of cells positive for CD31+ (Figure 4) and vWF+ (Figure 5), markers of endothelial cells, in the DPP4^D^-NC group than in DPP4-deficient rats with or without GCSF treatment. On the other hand, the numbers of these cells did not differ between DPP4-deficient rats with and without receiving GCSF after CLI induction. These findings indicate that GCSF treatment did not provide additional benefit in increasing the numbers of endothelial cells in the ischemic limb of the DPP4-deficient animals.

In WT Fischer rats, the numbers of CD31+ (Figure 4) and vWF+ (Figure 5) cells were significantly reduced after CLI induction but were substantially increased after GCSF treatment to levels comparable to those without receiving CLI procedure. These findings suggest that GCSF treatment provided an additional benefit in increasing the numbers of endothelial cells for angiogenesis in the ischemic limb.

**Figure 6:**
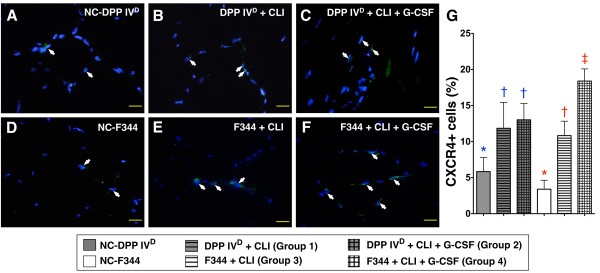
**Distribution of CXCR4+ cells in ischemic skeletal muscle on day 14 following CLI induction**. **A to F) **Immunofluorescent staining of CXCR4+ cells (white arrows) in ischemic skeletal muscle on post-CLI Day 14 with nuclei being counter-stained with DAPI (blue) (400x, *n *= 6 per group). **G) **In DPP4-deficient animals, significantly higher number of CXCR4+ cells in those with or without GCSF treatment than in DPP4^D^-NC, with no between the former two groups. * vs. †, *P *< 0.01. In WT Fischer rats, notably higher number of positively-stained cells in those with GCSF treatment than in those without and WT-NC, and higher in the WT-CLI group than in WT-NC. * vs. †, *P *< 0.0001. For comparison among the six groups, statistical analysis by one-way ANOVA. * vs. other groups, *P *< 0.0001. Symbols (*, †) indicate significant difference (at 0.05 level) by Tukey's multiple comparison procedure.

In both DPP4-deficient and WT Fischer rats, IF staining revealed that CXCR4+ (Figure 6) cells were significantly increased in DPP4^D^-CLI and WT-CLI rats compared with their respective controls (that is, DPP4^D^-NC and WT-NC). The numbers were further increased after GCSF treatment (that is, DPP4^D^-CLI-GCSF and WT-CLI-GCSF groups) (Figure 6). However, the amplitudes of increase were notably higher in WT Fischer rats compared to those in their DPP4-deficient counterparts.

**Figure 7:**
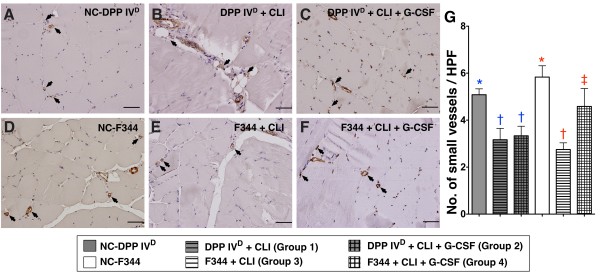
**Distribution of small vessels in ischemic skeletal muscle on Day 14 following CLI induction**. **A to F) **Quantification of small vessels (black arrows) (≤ 15 μm in diameter) through immunohistochemical staining of alpha-smooth actin (α-SMA). **G) **Number of vessels in ischemic muscle for each group (*n *= 6) on Day 14 following CLI induction. In DPP4-deficient animals, significantly lower number of small vessels in those with or without GCSF treatment than in DPP4^D^-NC, but similar between the former two groups. * vs. †, *P *< 0.03. In WT Fischer rats, significantly higher number of small vessels in WT-NC than in those with or without GCSF treatment, and higher in those with GCSF treatment than in those without. * vs. † vs. ‡, *P *< 0.001. Scale bars in right lower corner represent 50 μm. For comparison among the six groups, statistical analysis by one-way ANOVA. * vs. other groups, *P *< 0.001. Symbols (*, †, ‡) indicate significant difference (at 0.05 level) by Bonferroni's multiple-comparisons *post hoc *test.

In DPP4-deficient rats, the results of IHC staining demonstrated remarkably higher number of small vessels (defined as < 15.0 μm) in the DPP4^D^-NCgroup compared to those having received the CLI procedure with or without GCSF treatment that showed no difference among themselves (Figure 7). In WT Fischer rats, the number of small vessels was lower in the WT-CLI group than in WT-NC group and WT-CLI-GCSF group, and it was lower in WT-CLI-GCSF group than in WT-NC group (Figure 7). Of importance is that the number of small vessels was notably higher in WT-CLI-GCSF than in DPP4^D^-CLI-GCSF animals. Both IF and IHC findings demonstrated that WT Fischer rats exhibited better response to GCSF as reflected in the enhanced angiogenesis in the ischemic area compared with that in the DPP4-deficient rats after CLI induction. These findings could also explain the higher INBF ratio in the WT-CLI-GCSF group compared to that in the DPP4^D^-CLI-GCSF group.

### Femoral arterial vasorelaxation and NO release

**Figure 8:**
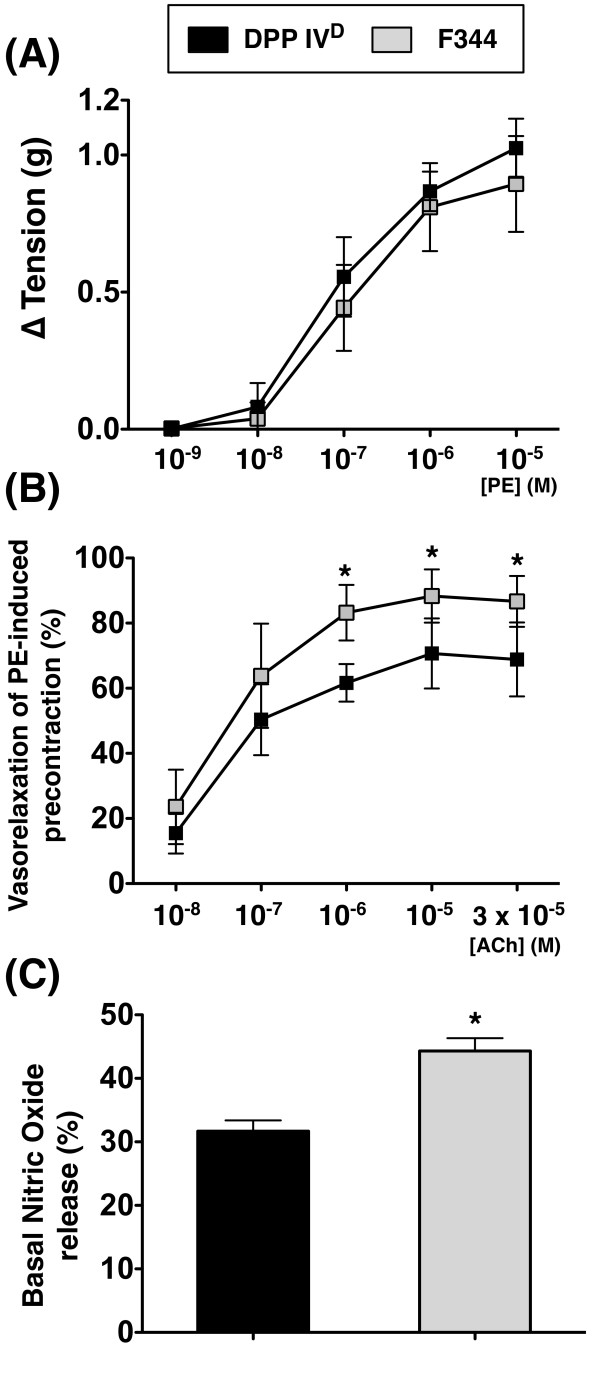
**Femoral arterial vasorelaxation and nitric oxide (NO) release following D-galactose administration**. Despite lack of a significant difference in vasoconstrictive response of femoral artery to phenylephrine (PE) between DPP4-deficient and Fischer 344 rats **(A)**, remarkably reduced vasorelaxation of femoral artery to achetylcholine (ACH) noted in DPP4-deficient rats compared to that in WT Fischer rats **(B)**. * vs. DPP4^D^, *P *< 0.01. **C) **Substantially reduced NO release from endothelium of femoral artery in DPP4-deficient rats compared to that in WT Fischer rats. * vs. DPP4^D^, *P *< 0.001 (*n *= 9 per group).

Alpha 1 adrenergic receptor agonist-induced vasoconstriction did not differ between DPP4-deficient rats and WT Fisher rats. However, vasorelaxation was more remarkably alleviated in DPP4-deficient rats than in WT Fischer rats (Figure 8B). Moreover, NO release from the endothelial cells of femoral artery was substantially reduced in DPP4-deficient rats compared to that in WT Fischer rats (Figure 8C).

### Numbers of EPCs in BM and SDF-1α levels in BM and circulation at 18 hour after CLI procedure

**Figure 9:**
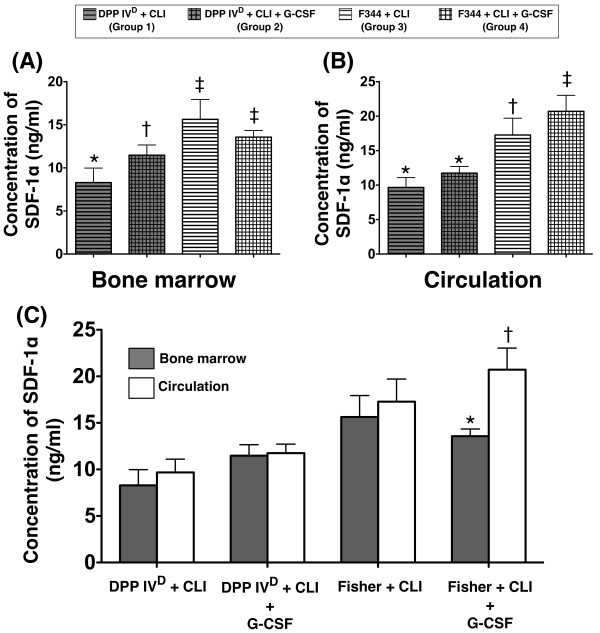
**ELISA analysis on SDF-1α levels in bone marrow (BM) and circulation at 18 h after CLI procedure**. **A) **Significantly higher BM SDF-1α level in Wild-type (WT) Fisher rats with or without GCSF treatment than in DPP4-deficient rats with or without GCSF treatment, and higher in DPP4-deficient rats with GCSF compared with those without. * vs. † vs. ‡, *P *< 0.001. Apparently higher expression in WT animals without than in those with GCSF treatment without statistical significance. **B) **Significantly higher circulatory SDF-1α level in WT-CLI-GCSF group than in other groups, notably higher in WT-CLI rats than in DPP4-deficient rats with or without GCSF treatment that showed no difference among themselves. * vs. † vs. ‡, *P *< 0.001. **C) **No difference in SDF-1α level between BM and circulation in those with or without GCSF treatment after CLI induction in DPP4-deficient rats, whereas higher level noted in circulation than BM in WT-CLI rats with further increase in circulation than in BM after GCSF treatment. * vs. † vs. ‡ vs. §, *P *< 0.0001. For comparison among the four groups, statistical analysis by one-way ANOVA. * vs. other groups, *P *< 0.0001. Symbols (*, †, ‡, §) indicate significant difference (at 0.05 level) by Bonferroni's multiple-comparisons *post hoc *test (*n *= 6 per group).

In DPP4-deficient rats, the SDF-1α level of the DPP4D-CLI group did not differ between BM and circulation (Figure 9C). Similarly, the concentration of this chemokine also showed no difference between BM and circulation in the DPP4^D^-CLI-GCSF group (Figure 9C). Furthermore, the circulating level of SDF-1α did not differ between DPP4-deficient rats with and without GCSF treatment after CLI induction (Figure 9B). However, the BM SDF-1α level was significantly higher in DPP4-deficiency rats with GCSF than in those without (Figure 9A).

In WT Fischer rats, the WT-CLI group had a higher circulatory SDF-1α level than that in BM, despite the lack of statistical significance (Figure 9C). On the other hand, SDF-1α level was significantly higher in circulation than in BM in the WT-CLI-GCSF group (Figure 9C). Furthermore, this biomarker in circulation was highest in the WT-CLI-GCSF group and significantly higher in WT-CLI than in DPP4-deficient animals with or without GCSF treatment (Figure 9B). Interestingly, this biomarker in BM was higher in WT-CLI than in WT-CLI-GCSF animals, although it showed no statistical significance (Figure 9A).

**Figure 10:**
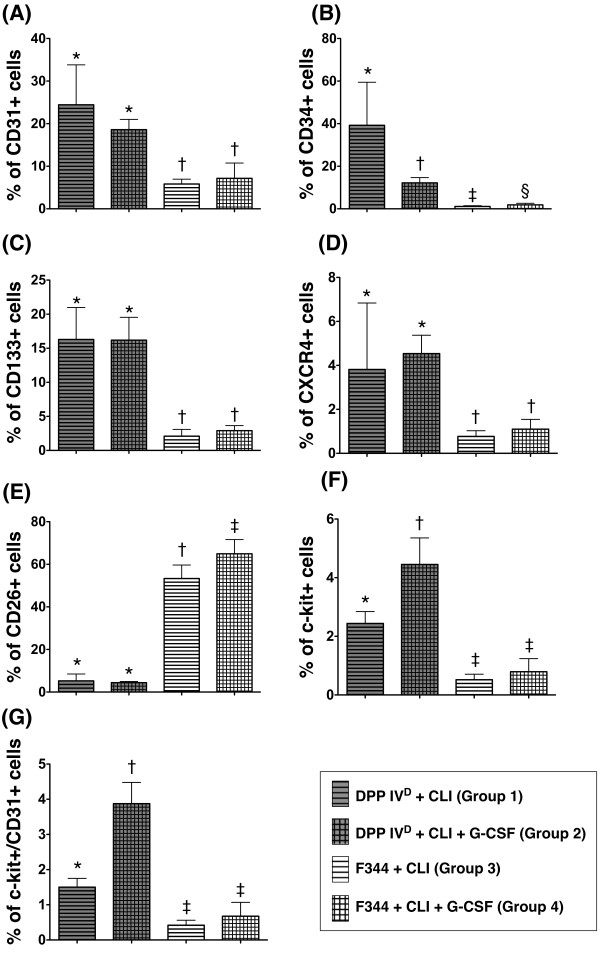
**Flow cytometric analysis of surface markers on bone marrow cells (CD31+, CD34+, CD133+, CXCR4+, C-kit, CD26) and identification of endothelial progenitor cells (EPCs) through double staining of C-kit/CD31 at 18 hour after CLI procedure**. Except for CD26+ cells, significantly higher numbers of EPCs in BM of DPP4-deficient rats regardless of GCSF treatment than that in wild-type Fischer rats with or without GCSF treatment. For comparison among the four groups, statistical analysis by one-way ANOVA. * vs. other groups, *P *< 0.0001. Symbols (*, †, ‡, §) indicate significant difference (at 0.05 level) by Bonferroni's multiple-comparisons *post hoc *test (*n *= 6 per group).

Compared with SDF-1α level in BM, the number of EPC in BM showed a reverse manner (that is, higher in DPP4-deficient than in WT animals) (Figure 10). These findings could explain the remarkably higher circulating number of EPC in WT Fischer rats compared to that in their DPP4-deficient counterparts in the setting of CLI with GCSF treatment.

## Discussion

To the best of our knowledge, this is the first study using DPP4-deficient rats to investigate the impact of inherent DPP4 enzyme deficiency on the circulating number of EPCs and angiogenic factors and blood flow in ischemic area in the setting of CLI. The results of the current study provided several striking implications. First, as compared with WT Fischer rats, DPP4-deficient rats had notably reduced ability for EPC mobilization into the circulation in response to CLI. Second, expressions of pro-angiogenic factors at both protein and molecular-cellular levels were significantly lower in DPP4-deficient animals compared to those in WT rats regardless of the presence or absence of GCSF treatment. Third, compared with WT Fischer rats, the capacity of basal NO release was impaired in the DPP4-deficient animals. Finally, the circulating SDF-1α level and the ratio of INBP, an index of circulatory functional recovery, were notably reduced in DPP4-deficient rats compared to those in WT Fischer rats by Day 14 after the CLI procedure.

### INBF ratio and number of vessels in ischemic area in DPP4-deficient and age-matched WT Fischer rats

One essential finding in the present study is that the number of small vessels, an index of neovasculogenesis, was remarkably lower in DPP4-deficient rats than in WT Fischer rats in ischemic area in response to GCSF treatment by post-CLI Day 14. In addition, another important finding in the current study is that, after GCSF treatment, the INBF ratio in ischemic area, an index of functional recovery [28], was more significantly reduced in DPP4-deficient rats than in WT Fischer rats. These findings imply that the recovery of blood flow was inferior in DPP4-deficient rats compared with that in WT Fischer rats after CLI induction with GCSF treatment.

### Changes in circulating EPC level at different time points in DPP4-deficient and age-matched WT Fischer rats

Previous studies have shown that inhibition of DPP4 enzyme either through ACEI or oral hypoglycemic agent enhanced circulating number of EPCs through prolonging the half-life of SDF-1α, thereby increasing its concentration in circulation [20,23,32]. Therefore, it is speculated that, as compared with WT Fischer rats, the circulating number of EPCs in DPP4-deficient rats should be notably higher not only at the baseline level but also in the ischemic setting. Surprisingly, except for CD34+ cells, the baseline level of circulating EPCs (that is, CD31+, CD133+, C-kit+) was not found to be significantly higher in DPP4-deficient rats compared to that in WT Fischer rats. One intriguing finding is that remarkably higher numbers of circulating EPCs at most time points were noted in WT Fischer rats compared to those in DPP4-deficient rats following CLI with or without GCSF treatment. The elevation in the circulating level of SDF-1α appears to account for the significantly higher circulating number of EPC in WT Fischer rats with CLI following GCSF treatment compared to that in other groups. Furthermore, these findings, in addition to supporting the proposal that the mobilization of EPCs from BM to circulation in response to CLI was poorer in DPP4-deficient rats than that in WT Fischer rats, could also partially explain the reduction in blood flow and number of small vessels in the former rather than in the latter.

### Molecular-cellular and protein levels of angiogenesis factors in DPP4-deficient and age-matched Fischer 344 rats after CLI procedure on post-CLI Day-14

Contrary to our hypothesis, the protein expressions of pro-angiogenic factors (that is, eNOS, VEGF, CXCR4 and SDF-1α) were significantly lower in DPP4-deficient rats compared to those in WT Fischer rats after CLI induction. Moreover, these factors were remarkably lower in the former than in the latter following GCSF treatment. Consistently, despite the lack of difference in the numbers of cells with angiogenic potential (CD31+, vWF+, CXCR4+, SDF-1α+) between DPP4-deficient and WT Fischer rats in the ischemic limb without treatment on IF staining and from Western analyses, IF staining revealed significantly higher numbers of these pro-angiogenic cells in the ischemic limb of WT Fischer rats compared to those in DPP4-deficient rats after GCSF treatment. Again, this apparently paradoxical finding implied that DPP4-deficient rats had suppressed GCSF-elicited angiogenesis in response to ischemic insult compared to their WT counterparts.

### Vasorelaxation and basal NO release from femoral arterial endothelial cells in DPP4-deficient and age-matched WT Fischer rats

The principal finding in the present study is that, as compared with WT Fischer rats, the endothelium-dependent vasodilatatory response of femoral artery was significantly reduced in DPP4-deficient rats. Since nitric oxide produced from endothelial NO synthase (eNOS) is the well-known endothelium-derived relaxing factor that participates in angiogenesis [2,28,33], the finding could be explained by the markedly decreased basal NO production from femoral artery endothelium in DPP4-deficient rats compared to that in WT Fischer rats in this study. Our findings, therefore, in addition to being supported by previous studies [2,33,34], could further explain the significantly reduced vessel density and blood flow in DPP4-deficient rats than in WT Fischer rats. On the other hand, impairment in NO production and its bioavailability has been demonstrated to be associated with accelerated vascular remodeling and pathogenesis of atherosclerosis [2,34].

### Possible explanation for the paradoxical findings of the present study

It has been reported that a higher circulatory stromal cell-derived factor (SDF)-1α concentration compared with that in BM creates a concentration gradient that has been demonstrated to play a crucial role in modulating EPC mobilization from BM into the circulation [35]. SDF-1α binds specifically to the receptor CXCR4 expressed on the surface of EPCs, especially CD34+ cells [36]. Therefore, SDF-1α acts as a principal chemokine that promotes the mobilization of EPCs from BM after MMP-9-mediated cleavage of membrane-bound C-kit-L [35,37]. Furthermore, SDF-1α, which is expressed in activated platelets, smooth muscle cells and ischemic cell/tissue, has been shown to be markedly increased in response to ischemic stimulus and mediates the recruitment of progenitor cells along the hypoxic gradients towards the ischemic zone [35]. On the other hand, since GCSF has been demonstrated to enhance the mobilization of stem cells and EPCs from BM into circulation [24,25], its impact on circulatory EPC concentration was investigated among the DPP4-deficiency and WT animals with and without CLI induction. The results of this study contradict our hypothesis that adult male DPP4-deficient rats have a higher circulating number of EPCs and better preserved endothelial function, angiogenesis capacity, and perfusion in ischemic area compared with their WT littermates. Of particular importance is that the ability of GCSF to mobilize EPC from BM to circulation appeared to be blunted in the DPP4-deficient rats compared to their WT littermates. This may be explained by a lack of significant reduction in BM SDF-1α concentration after GCSF administration in the DPP4-deficient animals (Figure 9). The relationship between BM SDF-1α concentration and circulatory EPC level has been reported when VEGF, an endothelium-derived pro-angiogenic chemokine similar to GCSF, was found to up-regulate MMP-9 concentration in BM that degrades BM SDF-1α, thereby creating a relatively high circulatory SDF-1α level to augment migration of EPCs from BM to circulation [20,21].

On the other hand, a paradoxical discrepancy between the findings from pharmacological and genetic model studies has been illustrated, for instance, in the studies of the role of p38 MAPK in cardiomyocyte hypertrophy using pharmacologic blockade of p38 [38,39] and transgenic animal models with reduced p38 signaling [40,41]. Although long-term pharmacologic blockade of p38 MAPK in spontaneously hypertensive rats was shown to reduce hypertrophy and augment survival [39], studies using transgenic animal models with over-expression of dominant-negative p38 demonstrated significantly aggravated cardiac hypertrophy following pressure-overload stimulation [40,41]. In the case of DPP4-deficient rats, one possible explanation for the discrepancy between the results of the current study and our original hypothesis may be the up-regulation of the other functional homologous family members of DPP, including DPP8 and DPP9 for which SDF-α is also the substrate, thereby leading to overcompensation. Such up-regulation in DPP activities by increasing expression of other functional homologous DPP has been previously reported in an experimental asthma DPP4-deficient rat model [42].

### Study limitations

This study has its limitations. First, the number of animals used for this study was relatively small. Nevertheless, the consistency of the results warrants further investigation into the interaction between the GCSF-DPP4 system and SDF-1α. Second, the present study did not really provide a mechanistic basis to delineate the poor angiogenic activity in DPP4-deficient rats. Thus, how the genetic defect reduces the angiogenic potential and relevant molecular factors remains uncertain. Third, since the BM and circulatory concentrations of other functionally homologous members of the DPP family were not studied, their possible compensatory roles in the setting of limb ischemia in DPP4-deficinent rats remain to be elucidated.

## Conclusions

**Figure 11:**
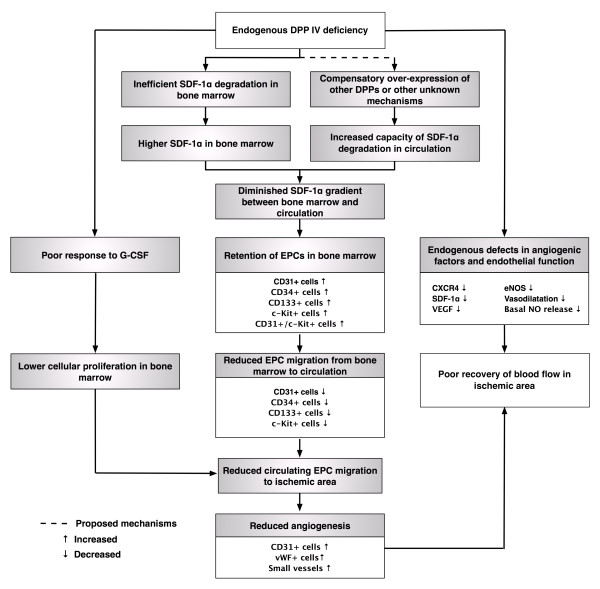
**Proposed mechanisms underlying the effects of DPP4-deficient on impairment of angiogenesis, endothelial function and circulating endothelial progenitor cell number based on the findings of the present study**. eNOS, endothelial nitric oxide synthase; EPC, endothelial progenitor cell; G-CSF, granulocyte colony-stimulating factor; NO, nitric oxide; SDF, stromal cell-derived factor; VEGF, vascular endothelial growth factor; vWF, von Willebrand factor.

Intrinsic angiogenic factors and basal nitric oxide release was impaired in DPP4-deficient rats, which were shown to exhibit inferior capacity of up-regulating angiogenesis factors, enhancing circulating number of EPCs, and augmenting blood flow in ischemic area after CLI compared to age-matched WT Fischer rats. The mechanisms we propose to be involved in the observed changes are illustrated in Figure 11.

## Abbreviations

ACEI: angiotensin converting enzyme inhibitor; ACh: acetylcholine; α-SMA: α-smooth muscle actin; BM: bone marrow; CAD: coronary artery disease; CLI: critical limb ischemia; CXCR4: C-X-C chemokine receptor type 4; DPP4: dipeptidyl peptidase-IV; ECL: enhanced chemiluminescence; ED: endothelial dysfunction; eNOS: endothelial nitric oxide synthase; EPC: endothelial progenitor cell; FITC: fluorescein isothiocyanate; GCSF: granulocyte colony-stimulating factor; HRP: horseradish peroxidase; IF: immunofluorescent; IHC: immunohistochemical; INBF: ischemic/normal blood flow; MNC: mononuclear cell; NC: normal control; NO: nitric oxide; PE: phenylephrine; SDF-1α: stromal cell-derived factor-1α; VEGF: vascular endothelial growth factor; vWF: von Willebrand factor; WT: wild-type.

## Competing interests

The authors declare that they have no competing interests of any sort, including commercial association, such as consultancies, stock ownership or other equity interests or patent-licensing arrangements.

## Authors' contributions

Cheuk-Kwan Sun and Steve Leu contributed equally to this work. Hsueh-Wen Chang and Hon-Kan Yip contributed equally to this work. CKS, SL and HKY participated in the design of the study, data acquisition and analysis as well as drafting of the manuscript. CKS, SL and JJS were responsible for the laboratory assay and troubleshooting. THT, YLC, HCS, SYC and HWC participated in data acquisition, analysis, interpretation and literature research. SFK, HWC, SL and HKY conceived of the study, participated in its design and coordination, and helped in drafting the manuscript. All authors read and approved the final manuscript.
